# Comparative study of lung cancer between smokers and nonsmokers: A real-world study based on the whole population from Tianjin City, China

**DOI:** 10.18332/tid/192191

**Published:** 2024-09-09

**Authors:** Wenlong Zheng, Guohong Jiang, Chong Wang, Luning Xun, Chengfeng Shen, Shuang Zhang, Hui Zhang, Qingxin Zhou, Meiqiu Xie, Xiaodan Xue, Dezheng Wang, Jun Lv

**Affiliations:** 1 Department of Epidemiology and Biostatistics, School of Public Health, Peking University, Beijing, China; 2 NCDs Preventive Department, Tianjin Centers for Disease Control and Prevention, Tianjin, China; 3 Peking University Center for Public Health and Epidemic Preparedness and Response, Beijing, China; 4 Key Laboratory of Epidemiology of Major Diseases, Peking University, Beijing, China

**Keywords:** lung cancer, non-smoker, histological subtypes, survival rate

## Abstract

**INTRODUCTION:**

The purpose of this study was to examine the prevalence, clinical characteristics, and changing trends of non-smokers with lung cancer (LC) based on data from a population-wide cancer registry in northern China.

**METHODS:**

The study used LC incidence and follow-up data from 2010 to 2019 from the Cancer Registry System of Tianjin city, which included 82769 cases. Trends in the incidence and proportion of non-smokers with LC were examined by joinpoint regression analysis. Life table and Cox survival analyses were used to calculate the survival rates and compare the death hazard ratios (HRs) in different groups, respectively.

**RESULTS:**

Among the 82769 new diagnosis cases of LC during 2010 to 2019, there were 34589 (41.8%) current smokers, 14913 (18.0%) ex-smokers, 28123 (34.0%) non-smokers, and 5144 (6.2%) unknowns. The proportion of non-smokers changed slightly from 2010 (35.36%) to 2019 (36.87%) (annual percentage change, APC= -0.01%, p>0.05). This proportion declined in men (2010 vs 2019; 22.06% vs 20.66%) and increased in women (2010 vs 2019; 53.02% vs 62.35%), and in the 0–44 years age group it showed an upward trend from 2015 to 2019 (APC=4.82%, 95% CI: 1.8–7.9). Compared with smokers with LC, non-smokers with LC were predominantly females (64.15% vs 27.26%), had a predominantly adenocarcinoma histological subtypes (76.71% vs 42.22%), and had a 20% lower risk of death than smokers (HR=0.80; 95% CI: 0.78–0.81).

**CONCLUSIONS:**

The proportion of non-smokers with LC was relatively high in northern China, with an increasing trend in the proportion of females and younger age groups. Non-smokers with LC had different epidemiological and clinical characteristics compared with smokers with LC.

## INTRODUCTION

Lung cancer (LC) was the second most commonly diagnosed cancer and the leading cause of cancer death worldwide, with an estimated 2.2 million new cancer cases and 1.8 million deaths in 2020^1^, of which approximately 37.0% of new LC cases and 39.8% of deaths occurred in China^1^. Smoking is known to be a major risk factor for LC, yet approximately 15–20% of males and >50% of females with LC were non-smokers^2^. Previous studies showed that never smokers with LC had special epidemiological and clinical features^3^, but some results were inconsistent , such as the diagnosis age. With the continuous development of the tobacco control campaign, the proportion of non-smokers with LC was still increasing^4,5^, which was estimated would become the predominant type of LC in the next decade. Therefore, it is very important to study the characteristics and changing trends of nonsmokers with LC for the development of prevention and control strategies, especially in China, where the female non-smoking rate is very high.

Previous studies about non-smokers with LC were most about cases from several hospitals, rarely studies from population-based registry. In this study, we will use data from the Cancer Report System of Tianjin city, which covers the whole population for up to 10 years. Thus, we can compare the characteristics and changing trends of smoking and non-smoking LC patients of the whole population during 10 years.

## METHODS

### Data sources

*Data collection*

LC incidence data (ICD10: C33-C34, including trachea and bronchus) were from the Tianjin Tumor Registration System which covers the whole population. Medical institutions at all levels in the city were required to report new cancer cases through network reporting system, and quality control was conducted through a three-levels of review by medical institutions, county CDCs, and municipal CDC. The information of tumor incidence cases was checked and supplemented mutually based on the International Cancer Registration Association, The Ninth Volume of Cancer Incidence on Five Continents and the Chinese Cancer Registration Guidance Manual^6^. Relevant variables were extracted from the patients’ hospital medical records, clinical course records, clinical examination, and clinical imaging studies. Baseline characteristics included: sex, age at diagnosis, marital status, occupation, and smoking history. Tumor related information was: pathological type, diagnosis basis, and sub-site of lung cancer patients. The death outcomes of LC patients were followed up through the Tianjin Resident Cause of Death Surveillance System, which covered all death cases of the whole population in Tianjin city^7^, using unique ID numbers.

*Smoking status*

Current smokers were defined as those who had smoked at least 100 cigarettes (or equivalent amount of tobacco) in their lifetime and were still smoking at the time of the survey. Ex-smokers were those who had smoked at least 100 cigarettes (or equivalent amount of tobacco) in their lifetime, but were not smoking at the time of the survey. Non-smokers were those who either had never smoked at all or had never been daily smokers and smoked less than 100 cigarettes (or equivalent amount of tobacco) in their lifetime^8^.

*Histological subtypes*

The histological subtypes of LC were defined according to the international classification system^9^. LC cases were sub-divided into squamous cell carcinoma (SCC) (M8070, M8083, M8072, M8074, or M8076), adenocarcinoma (ADC) (M8140, M8144, M8211, M8230, M8250, M8251, M8255, M8260, M8333, M8549, M8550, M8574, or M8576), small cell carcinoma (SCC) (M8041, or M8045), large cell carcinoma (LCC) (M8012, M8013, M8246, or M8310), adeno-squamous carcinoma (ASC) (M8560).

### Statistical analysis

The data are expressed as frequency and percentage for categorical variables. Qualitative variables were analyzed using the chi-squared test, such as comparisons of data between current smokers, non-smokers, and ex-smokers, and continuous variables such as diagnosis age were analyzed using the Kruskal-Wallis test using Statistical Package for Social Sciences software (version 21.0, SPSS, IBM Corp. Armonk, NY, US). All statistical tests were two-sided, and p<0.05 was considered statistically significant.

### Trends analysis

Segi’s world standard population age composition was used to calculate the age-standardized incidence rate (ASR) of cancers in Tianjin. The trends of incidence rates and proportions of non-smokers with LC, which include the annual percentage change (APC) and 95% CI, were examined by joinpoint regression analysis (using Joinpoint Statistical Software, Version4.3, April 2016, Statistical Methodology and Applications Branch, Surveillance Research Program, National Cancer Institute, US)^10^.

### Survival analysis

Survival time measures the time from the date of diagnosis to death^11^. Life table was used to calculate the 1-year and 5-year survival rates and compare the median survival time in different groups. The Cox proportional hazards regression model (forward stepwise, likelihood ratio test) with hazard ratios (HRs) and 95% confidence intervals (CIs) calculated, was used to conduct multivariate analysis of factors (including sex, age group, histology and smoking status) influencing survival time. As shown in Supplementary file Figure 1, log-log survival plots were used to check the proportional hazards assumption. The assumptions of proportionality were met for the Cox models. Statistical Package for Social Sciences software (version 21.0, SPSS, IBM Corp. Armonk, NY, US) was used to conduct all statistical analyses. All statistical tests were two-sided, and p<0.05 was considered statistically significant.

## RESULTS

### Incidence rate of LC and proportion of nonsmokers

*Incidence rate*

From 2010 to 2019, a total of 82769 new cases of LC were reported in Tianjin city. The total crude incidence rate of LC showed an upward trend from 2010 to 2017 (APC=1.62%; 95% CI: -0.2–23.4), rising from 78.59/100000 to 84.68/100000, and a downward trend from 2017 to 2019 (APC= -6.71%, 95% CI: -18.5–6.8), but none of the trends was statistically significant (p>0.05). From 2010 to 2019, the total age-standardized incidence rate (ASR) of LC showed a decreasing trend, from 50.91/ 100000 in 2010 to 40.45/ 100000 in 2019 (APC= -1.5%, 95% CI: -2.7–0.3, p<0.01). ASR showed a stable trend in males (APC= -0.9%, 95% CI: -2.1–0.4, p>0.05) and a significant downward trend in females (APC=-2.5%, 95% CI: -3.8 – -1.3, p<0.01). Analysis by age group showed that both men and women showed a significant downward trend in the 60–74 years age group, while the change trend in other age groups did not have statistical significance (Table 1).

**Table 1:** The incidence rates (% per 100000) of lung cancer and changing trends, by sex and age group, 2010–2019

Year	Total crude rate	Total standard rate	Standard rate of men	Standard rate of	0–44 years women	45–59 years	60–74 years	≥75 years
2010	78.59	50.91	59.58	42.24	2.62	56.22	278.40	508.30
2011	75.76	47.86	57.78	37.95	2.16	54.56	257.41	488.99
2012	77.53	48.20	57.51	38.88	2.23	53.34	260.88	494.42
2013	84.23	51.44	62.23	40.64	2.87	61.20	261.50	543.43
2014	77.76	45.70	55.04	36.35	2.09	53.60	235.41	488.66
2015	84.01	47.68	58.64	36.73	2.36	56.90	237.46	524.61
2016	85.31	47.42	57.65	37.18	2.60	61.01	240.03	480.56
2017	84.68	47.93	59.16	36.69	2.40	59.82	234.70	524.44
2018	81.83	45.62	57.23	34.01	2.29	56.12	227.64	491.18
2019	73.71	40.45	50.73	30.16	2.09	50.19	206.35	418.27
APC (95% CI)	2010–2017 1.62 (-0.2–34) 2017–2019 -6.71 (-18.5–6.8)	-1.5 (-2.7 – -0.3)	-0.9 (-2.1–0.4)	-2.5 (-3.8 – -1.3)	-1.0 (-3.7–1.9)	-0.1 (-1.8–1.7)	-2.6 (-3.4 – -1.7)	-0.9 (-2.6–0.8)

APC: annual percentage change.

*Percentage of non-smokers*

**Figure 1:**
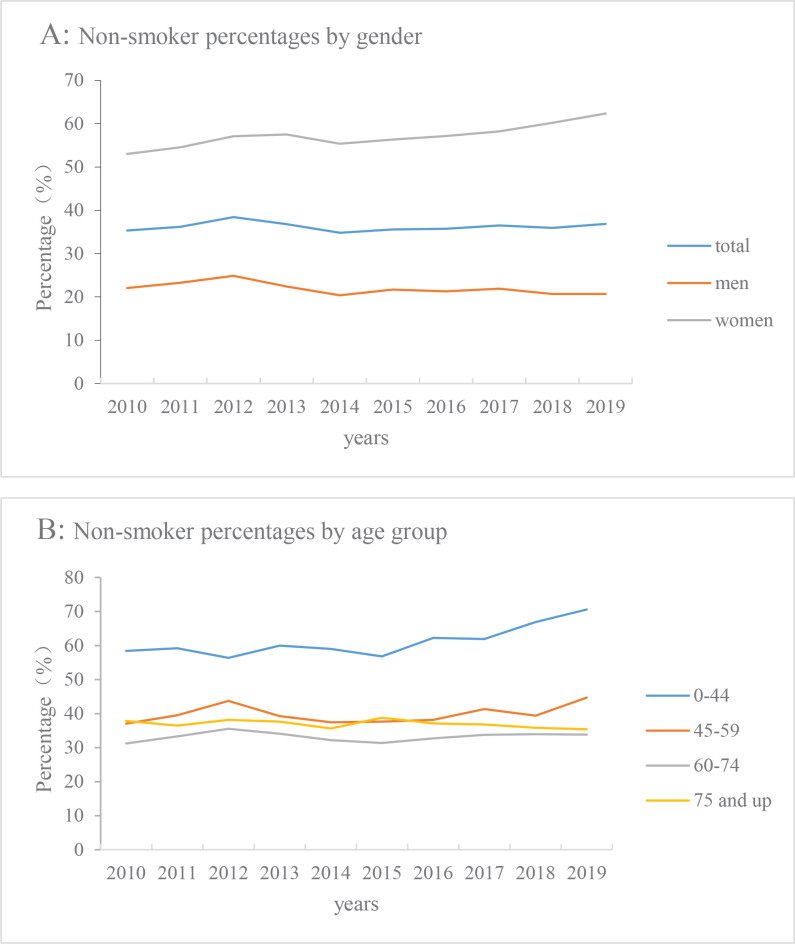
Percentage of non-smokers with LC, by gender and age group (2010–2019)

Among 82769 new LC cases, 34589 (41.8%), 14913 (18.0%), 28123 (34.0%) and 5144 (6.2%) were current smokers, ex-smokers, non-smokers and unknowns, respectively. The proportion of non-smokers in newly diagnosed LC patients was stable from 2010 to 2019, which was 35.36% in 2010 and 36.87% in 2019 (APC= -0.01%, p>0.05). (Figure 1A) The proportion of non-smokers in the 0–44 years age group was the highest, followed by the 45–59 and ≥75 years age groups, the 60–74 years age group had the lowest proportion. In the 0–44 years age group, the proportion of non-smokers was stable during 2010–2015 (APC= -0.02%, 95% CI: -2.3–2.0), and rose during 2015–2019 (APC=4.82%, 95% CI: 1.8–7.9). From 2010 to 2019, the 45–59, 60–74 and ≥75 years age groups all showed stable trends (Figure 1B).

Gender analysis showed that the proportion of nonsmoking male LC patients had decreased (2010 vs 2019; 22.06% vs 20.66%) (APC= -1.3%, 95% CI: -2.6–0.1), while the proportion of non-smoking female LC patients was higher than males and showed an increasing trend (2010 vs 2019; 53.02% vs 62.35%) (APC=1.4%, 95% CI: 0.8–2.0) (Figure 1A).

### Epidemiological and clinical characteristics of non-smokers and smokers with LC

*Sex characteristics*

Current smokers and ex-smokers with LC were predominantly male, accounting for 72.47% and 72.67%, respectively. The majority of non-smokers with LC were female, accounting for 64.15% (Table 2).

**Table 2:** Characteristics of lung cancer patients, by smoking status

*Characteristics*	*Current smoker n (%)*	*Former smoker n (%)*	*Never smoker n (%)*	*Unknown n (%)*
**Total**	34589 (41.8)	14913 (18.0)	28123 (34.0)	5144 (6.2)
**Sex**				
Men	25159 (72.74)	10837 (72.67)	10081 (35.85)*	2929 (56.94)*
Women	9430 (27.26)	4076 (27.33)	18042 (64.15)*	2215 (43.06)*
**Age** (years), median (IQR)				
Total	69 (61–76)	72 (65–79)*	69 (60–78)*	65 (57–74)*
Men	66 (59–74)	70 (63–78)*	71 (62–79)*	65 (57–73)*
Women	74 (68–79)	76 (70–81)*	68 (60–77)*	65 (57–75)*
**Age group** (years)				
0–44	467 (1.35)	104 (0.70)*	902 (3.21)*	312 (6.07)*
45–59	6739 (19.48)	1709 (11.46)*	5574 (19.82)	1349 (26.22)*
60–74	16646 (48.13)	6975 (46.77)*	11744 (41.76)*	2303 (44.77)*
≥75	10737 (31.04)	6125 (41.07)*	9903 (35.21)*	1180 (22.94)
**Region**				
Urban	21023 (60.78)	9287 (62.27)*	15435 (54.88)*	3168 (61.59)
Rural	13566 (39.22)	5626 (37.73)*	12688 (45.12)*	1976 (38.41)
**Histology**	12104 (44.8)	4854 (18.0)	9263 (34.3)	799 (3.0)
SCC	4274 (35.31)	1598 (32.92)*	1161 (12.53)*	158 (19.77)*
ADC	5110 (42.22)	2282 (47.01)*	7106 (76.71)*	538 (67.33)*
SCLC	2322 (19.18)	834 (17.18)*	810 (8.74)*	89 (11.14)*
LCC	180 (1.49)	65 (1.34)	62 (0.67)*	6 (0.75)
ASC	218 (1.80)	75 (1.55)	124 (1.34)*	8 (1.00)

* The difference was statistically significant compared with the current smoking group, p<0.05. Data between current smokers and non-smokers or ex-smokers were compared using the chi-squared test for qualitative variables including sex, age group, region, histology, and Kruskal-Wallis testing for diagnosis age.

*Diagnosis age*

For males, the median diagnosis age of non-smokers with LC (71 years) was higher than that of current smokers (66 years) (p<0.05). In contrast, female non-smokers with LC had a mean diagnosis age that was lower (68 years) than that of current smokers (74 years). The average age of diagnosis was higher in both male and female ex-smokers than in smokers (Table 2).

*Histology*

The proportion of ADC in non-smokers with LC (76.71%) was higher than that in current smokers with LC (42.22%). The proportions of SCC (12.53% vs 35.31%), SCLC (8.74 vs 19.18%), LCC (0.67% vs 1.49%) and ASC (1.34% vs 1.80%) in non-smokers with LC were all lower than those of current smokers with LC (Table 2).

*Survival time*

**Figure 2:**
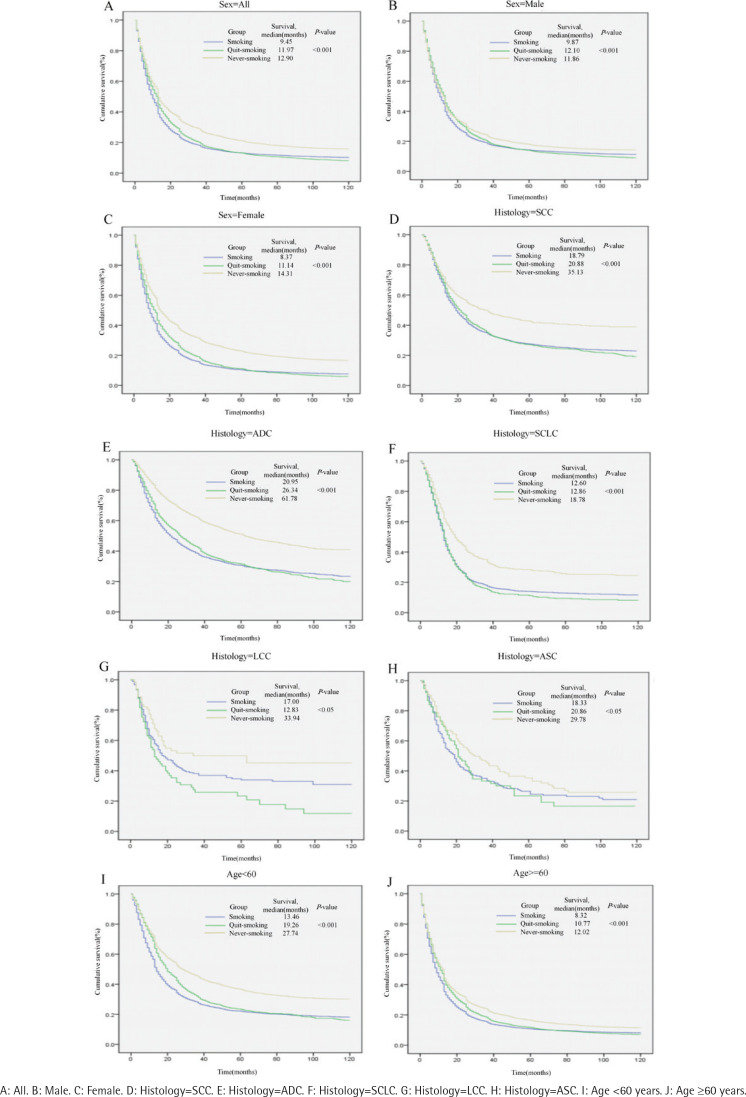
Cumulative survival rate of lung cancer patients, by smoking status

During the follow-up period, 66774 of 82769 lung cancer cases resulted in death. The 1-year and 5-year survival rates of non-smokers with LC were higher than those of current smokers, 53.8% vs 42.5% and 20.7% vs 13.0%, respectively. In addition, the survival rates of non-smokers with LC were all higher than those of current smokers across gender, age groups, and pathological types, as shown in Table 3. Cox survival analysis showed that both non-smokers (HR=0.80, 95% CI: 0.78–0.81) and ex-smokers (HR=0.85; 95% CI: 0.84–0.87) had lower risks of death than smokers. The difference in survival time between smokers and non-smokers with lung cancer was more significant among females and those aged <60 years (Table 4, Figure 2).

**Table 3:** Survival rates of lung cancer patients, by smoking status

*Characteristics*	*Median survival time (years)*			*Survival rate (%) 1-year*			*Survival rate (%) 5-year*		
	*Current*	*Former*	*Never*	*Current*	*Former*	*Never*	*Current*	*Former*	*Never*
**Overall**	0.88	0.99*	1.26*	42.5	48.7	54.8	13.0	12.9	20.7
**Sex**									
Men	0.90	1.00*	0.99*	43.5	49.3	48.2	14.1	13.7	17.6
Women	0.84	0.96*	1.43*	39.9	47.1	56.9	10.2	10.9	22.5
**Age** (years)									
0–44	1.59	2.15*	2.90*	60.8	79.8	75.6	26.1	29.7	42.0
45–59	1.27	1.74*	2.39*	54.5	67.1	70.4	21.6	22.8	35.2
60–74	0.93	1.25*	1.55*	45.4	54.6	59.3	14.2	16.6	24.8
≥75	0.72	0.79*	0.79*	29.8	36.3	35.9	5.2	5.9	6.4
**Histology**									
Unknown	0.75	0.85*	0.87*	33.0	41.1	42.3	6.2	7.0	8.5
SCC	1.71	1.82	2.93*	65.0	67.0	71.3	27.3	26.9	42.3
ADC	1.84	2.27*	5.00*	64.5	70.2	83.0	30.1	31.3	50.5
SCLC	1.12	1.15	1.70*	52.2	54.0	65.7	14.0	11.0	27.8
LCC	1.62	1.19	2.95	60.0	53.8	67.7	33.8	23.3	49.9
ASC	1.57	1.86	2.55*	61.0	70.7	71.8	24.8	23.0	35.6

* The difference was statistically significant compared with the current smoking group by multiple pairwise comparisons method, p<0.05.

**Table 4:** Multivariable adjusted hazard ratio for deaths of LC, by different characteristics

*Characteristics*	*Deaths/LC cases*	*AHR (95% CI)*
**Sex**		
Men ®	40115/49006	1
Women	26659/33763	0.90 (0.89–0.92)
**Smoking**		
Current ®	30180/34589	1
Former	13070/14913	0.85 (0.84–0.87)
Never	22214/28123	0.80 (0.78–0.81)
**Age** (years)		
0–44 ®	969/1785	1
45–59	10451/15371	1.27 (1.19–1.35)
60–74	29446/37668	1.56 (1.46–1.66)
≥75	25908/27945	2.14 (2.01–2.19)
**Histology**		
Uncertain ®	49270/55749	1
SCC	5027/7191	0.45 (0.44–0.47)
ADC	8606/15036	0.39 (0.38–0.40)
SCLC	3355/4055	0.71 (0.68–0.74)
LCC	205/313	0.46 (0.40–0.52)
ASC	311/425	0.51 (0.45–0.57)

AHR: adjusted hazard ratio. ® Reference categories.

The survival rates of different histological subtypes of lung cancer were also different. It was the lowest in small cell carcinoma, and higher in adenocarcinoma and large cell carcinoma than other types, as shown in Table 3. Cox survival analysis showed that women had a lower risk of death than men (HR=0.90; 95% CI: 0.89–0.92). The hazard ratios were 1.27 (95% CI: 1.19–1.35), 1.56 (95% CI: 1.46–1.66), and 2.14 (95% CI: 2.01–2.19) in the 45–59, 60–74, and ≥75 years age groups, respectively, compared with the 0–44 years age group. The risk of death was significantly lower in lung cancer patients with pathological type reported than uncertain cases, and the hazard ratio was 0.71 (95% CI: 0.68–0.74) for small cell carcinoma, 0.51 (95% CI: 0.45–0.57) for squamous adenocarcinoma, 0.46 (95% CI: 0.40–0.52) for large cell carcinoma, 0.45 (95%CI: 0.44–0.47) for squamous cell carcinoma, and 0.39 (95% CI: 0.38–0.40) for adenocarcinoma in descending order compared to LC patients with no pathological type reported (Table 4).

## DISCUSSION

Based on population-wide registry data, we found that the proportion of non-smoking LC patients was relatively high in northern China. The epidemiological and clinical characteristics of non-smokers with LC were much more different from those of smokers. About 15–20% males and >50% females in global LC cases were non-smokers^2^. Non-smoking LC accounted for a relatively high proportion in Asia (31%), while the proportion of non-smokers in Europe and America was relatively low, accounting for 18.9%^12^. Siegel et al.^13^ reported that 12.5% LC cases in seven US states from 2011 to 2016 were non-smokers, 9.6% among men and 15.7% among women^13^. The proportion of non-smokers with LC in this study was slightly higher than in previous studies in Asia and much higher than that in the United States, indicating that non-smokers with LC is a more prominent problem in China. Previous reports had shown that the proportion of non-smokers with LC was on the rise in the UK and the US^4,5^. The results of this population-based study in northern China showed that the proportion of non-smokers with LC had a significant upward trend in women and in the young age group (0–44 years). This may mainly be related to a significant decrease in smoking rates. Previous study showed that the smoking rates of younger adults (20–39 years) decreased the most, from 35.3% in 1996 to 17.2% in 2015; and also for women, from 17.3% in 1996 to 4.5% in 2015^14^. But the incidence rate of LC in the 0–44 years group did not have a significant decreasing trend, indicating that the disease burden of non-smokers with LC in this age group is on the rise and requires more attention. Exposure to secondhand smoke and indoor and outdoor air pollution are important risk factors for non-smoking lung cancer. More effective preventive measures should be taken to reduce risk factors^15^.

Previous studies had shown that non-smokers with LC had different epidemiological and clinical characteristics from smokers. Smokers with LC were predominantly male, while non-smokers with LC were predominantly female^3^, which was consistent with the results of the present study. However, this did not mean that women had a higher incidence rate of non-smoking LC than men. Among the few studies on the incidence of LC in non-smokers, Wakelee et al.^16^ analyzed data from six large cohort studies in the United States and showed that the age-standardized incidence rate of LC in female non-smokers aged 40–79 years ranged from 14.4 to 20.8 per 100000 person-years, which was significantly higher than that of males (4.8–13.7/100000 person-years). Thun et al.^17^ used data from 13 large cohort studies and 22 cancer surveillance systems around the world to describe the current status of LC incidence and mortality in non-smokers. The study pointed out that the standardized incidence rate of LC in non-smoker females aged >40 years in Asia was 14.8 per 100000. The rate for males was 12.7/100000, and there was no significant difference between males and females^17^. The main reason for the higher proportion of non-smokers with LC among females than among males may be the lower incidence rate of smoking among females than among males.

Currently, conclusions regarding the age of first diagnosis of smoking and non-smoking LC patients are inconsistent. Some researches supported that non-smokers have a higher age of diagnosis^18,19^, some had come to the opposite conclusion^20,21^, and others had concluded that there is no significant difference in the age of first diagnosis between the two groups^22,23^. The results of meta-analysis showed that proportion of non-smokers was higher than that of smokers among young LC patients (≤45 years)^7^. Using population-wide tumor registry data, our study showed that the average age at diagnosis of lung cancer was higher in male non-smokers than in current smokers, while the opposite was true for females. Previous study also showed non-smoking cases tended to be younger compared with the smoking cases in Chinese women^24^. The reasons for this gender difference needs to be further studied. This could be due to differences in lung cancer type or etiology, or to smoking patterns at different ages, given that older women had a higher smoking rate than the young women (about ten times) in Tianjin city^25^. This study also found that both men and women who quit smoking were diagnosed with lung cancer for the first time at a higher average age than current smokers, suggesting that quitting smoking has significant health benefits.

The study showed that the proportion of SCC LC decreased successively in current smokers, ex-smokers and non-smokers, while the proportion of ADC increased successively, indicating that the pathological type of LC was closely related to smoking status, which was consistent with previous studies^26^. Studies had shown that there was a significant dose-response relationship between the proportion of SCC and smoking^27^. The proportion of SCC in male lung cancer patients with smoking quantity >20 packs/year and <10 packs/year was 44.41% and 32.21%, respectively. In recent years, the histological subtype of LC has been reported in many countries including China^28-30^, where ADC has been more frequently diagnosed than SCC. The shift in LC trends by histological type that occurred over the last several decades had been hypothesized to be due to changes in cigarette composition and design^31,32^.

Consistent with previous studies, our study found that the proportion of adenocarcinoma in both smokers and non-smokers showed a rapid upward trend, and the increasing rise in non-smokers with LC was even greater than in smokers (Supplementary file Figure 2), indicating that the change of smoking status now may not be the main factor affecting the change of LC pathological types. It was found that the proportion of lung adenocarcinoma was closely related to the pathological detection rate in this study (Supplementary file Figure 3). In the initial round of screening of study participants in the National Lung Screening Trial (NLST), LC screening with low-dose computed tomography (LDCT) identified a higher proportion of adenocarcinoma than squamous cell carcinoma^33^. The gradual increase in the rate of pathological detection may be an important reason for the increase in the proportion of adenocarcinoma.

Most studies suggest that the survival rate of LC in non-smokers was higher than that in smokers^16,19^. Japuntich et al.^34^ followed up 5575 patients with LC and found that non-smokers had a 29% lower risk of death than smokers (HR=0.71; 95% CI: 0.57–0.89). However, the Subramanian et al.^35^ study showed no significant difference in 5-year survival between smokers and non-smokers in 254 matched pairs of lung cancer patients. The results of this study based on the data analysis of the whole population, support that the survival rate of LC in non-smokers is higher than that in smokers, and the longer time follow-up, the greater the difference in death risk between the two, which proves that non-smoking LC has a better prognosis than smoking LC. At the same time, the survival rate of ex-smokers was significantly higher than that of current smokers, indicating that quitting smoking has obvious health effects.

### Limitations

The study has some limitations. First, all cases were from one city of northern China, so the results may not have national representativeness, and also need to be carefully generalize to other countries. Second, due to monitoring data limitations, this study only collected smoking status data from lung cancer patients and did not collect data on smoking volume, secondhand smoke exposure levels etc., which affect the in-depth analysis. Third, a certain percentage of LC cases (6.2%) did not accurately report smoking status, and the proportion of non-smokers and smokers with LC might be affected.

## CONCLUSIONS

Based on population-wide data, we found that the proportion of non-smokers with LC was relatively high and there was an increasing trend in women and younger age groups in northern China. Non-smokers with LC had different epidemiological and clinical characteristics compared with smokers in gender, diagnosis age, histology and survival time.

## Supplementary Material



## Data Availability

The data supporting this research are available from the authors on reasonable request.
