# Supplementary material for: Comparative study of lung cancer between smokers and nonsmokers: A real-world study based on the whole population from Tianjin City, China
Source: Tob Induc Dis. 2024 Sep 9;22:10.18332/tid/192191. doi: 10.18332/tid/192191 (PMC11382349; doi:10.18332/tid/192191)
Supplement: Supplementary file 1 [file TID-22-154-s1.pdf]

A. LC of current smokers

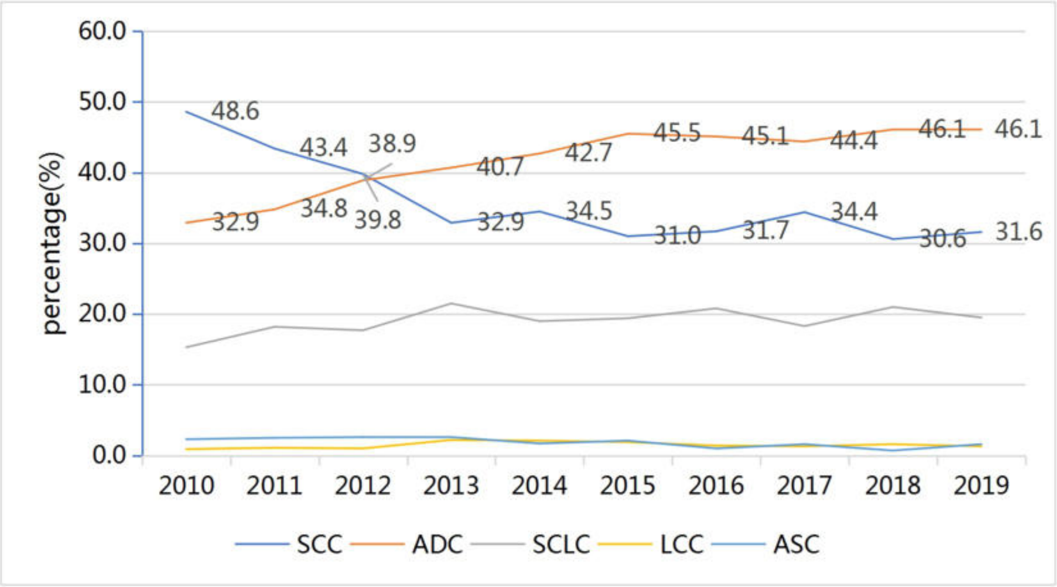

B. LC of non-smokers

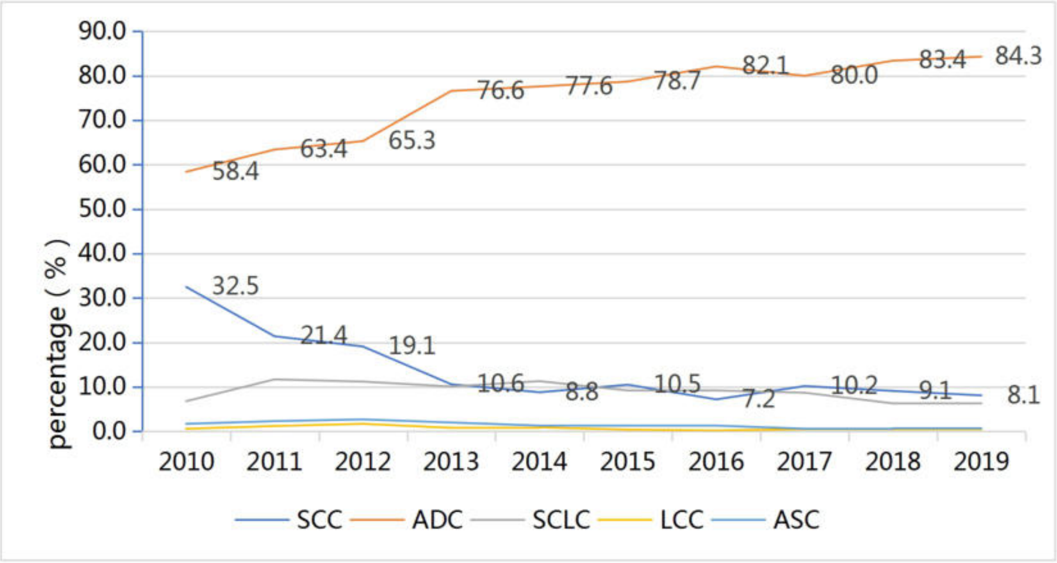

Supply Figure1 The percentages of histological subtypes of lung cancer from the year of 2010 to 2019

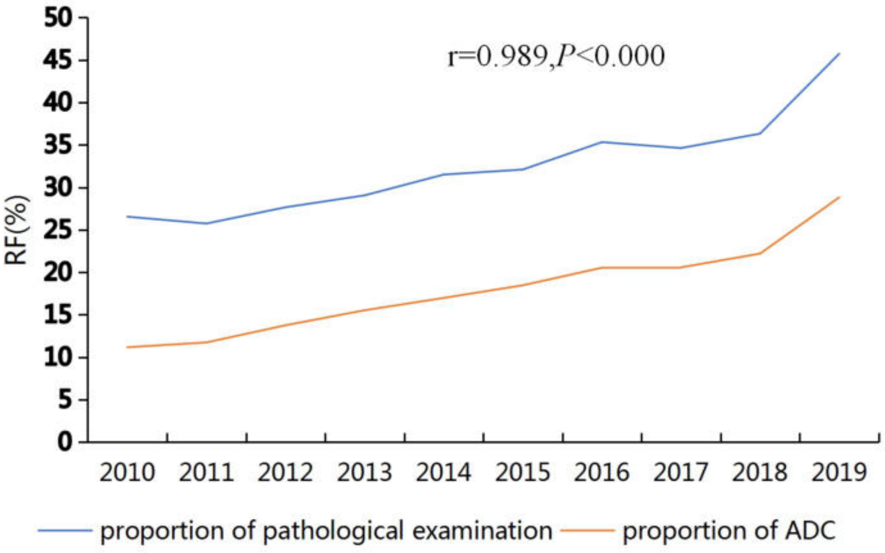

Supply figure2 The correlation of the proportion of pathological and the proportion of ADC of lung cancer

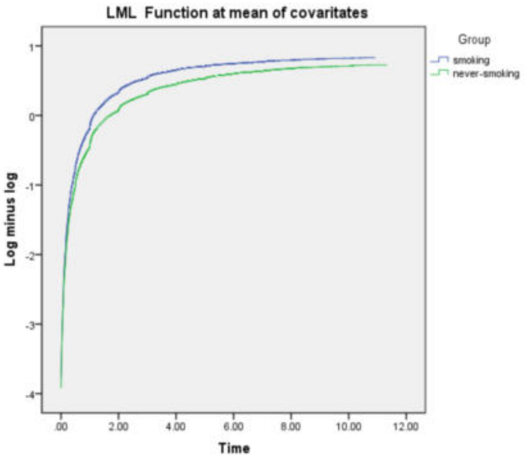

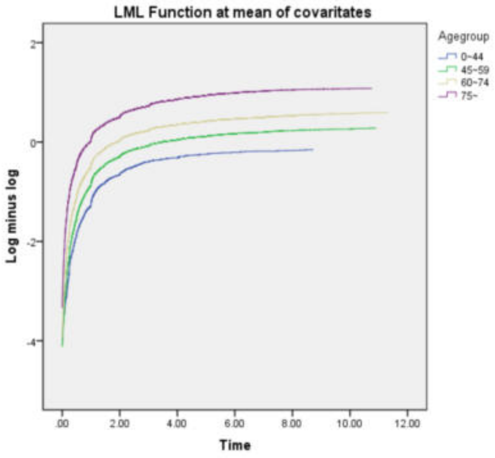

Supply Figure3 Plot to check proportional hazards assumption of the Cox model.

©2024 Zheng W. et al.
